# Vpu-dependent block to incorporation of GaLV Env into lentiviral vectors

**DOI:** 10.1186/1742-4690-7-4

**Published:** 2010-01-26

**Authors:** Ilias Christodoulopoulos, Magali E Droniou-Bonzom, Jill E Oldenburg, Paula M Cannon

**Affiliations:** 1 Department of Molecular Microbiology and Immunology, Keck School of Medicine, 2011 Zonal Avenue, University of Southern California, Los Angeles 90033, California, USA

## Abstract

**Background:**

The gibbon ape leukemia virus (GaLV) Env protein mediates entry into a wide range of human cells and is frequently used to pseudotype retroviral vectors. However, an incompatibility exists between GaLV Env and lentiviral vectors that results in decreased steady-state levels of the mature GaLV Env in cells and prevents its incorporation into lentiviral vector particles.

**Results:**

We identified the HIV-1 Vpu protein as the major cause of the depletion in GaLV Env levels that occurs when lentiviral vector components are present. This activity of Vpu targeted the mature (cleaved) form of the GaLV Env that exists within or beyond the trans-Golgi. The activity required two conserved phospho-serines in the cytoplasmic tail of Vpu that are known to recruit β TrCP, a substrate adaptor for an SCF E3 ubiquitin ligase complex, and could be blocked by mutation of lysine 618 in the GaLV Env tail. Moreover, the Vpu-mediated decrease of GaLV Env levels was inhibited by the lysosomal inhibitor, bafilomycin A1. Interestingly, this activity of Vpu was only observed in the presence of other lentiviral vector components.

**Conclusions:**

Similar to the mechanism whereby Vpu targets BST-2/tetherin for degradation, these findings implicate β-TrCP-mediated ubiquitination and the endo-lysosomal pathway in the degradation of the GaLV Env by lentiviral vector components. Possibly, the cytoplasmic tail of the GaLV Env contains features that mimic *bona fide *targets of Vpu, important to HIV-1 replication. Furthermore, the lack of effect of Vpu on GaLV Env in the absence of other HIV-1 proteins, suggests that a more complex interaction may exist between Vpu and its target proteins, with the additional involvement of one or more component(s) of the HIV-1 replication machinery.

## Background

Both human immunodeficiency virus type 1 (HIV-1)-based lentiviral and murine leukemia virus (MuLV)-based retroviral vectors are used clinically in human gene therapies. However, lentiviral vectors offer an advantage over the more widely used retroviral vectors in their ability to transduce non-dividing cells in a range of organs [[1-3], reviewed in [4]]. A key feature of both lentiviral and retroviral vectors is their ability to incorporate heterologous fusion proteins [reviewed in [5]], in particular the broadly-tropic vesicular stomatitis virus (VSV) G protein [6,7], in a process known as pseudotyping. This allows user-defined host targeting of these vectors, depending on their downstream purpose.

The Gammaretrovirus gibbon ape leukemia virus (GaLV) Env protein, which has been shown to use a sodium-dependent phosphate transporter protein (Pit-1) as its receptor [8,9], is frequently used to pseudotype retroviral vectors, due to its broad host range and high efficiency at transducing certain human cell types [10,11]. Previously we and others reported that although the GaLV Env could efficiently pseudotype retroviral vectors, it was not able to pseudotype lentiviral vectors. This was in marked contrast to the closely related amphotropic MuLV Env protein which could efficiently pseudotype both vector particles [12,13]. This phenomenon could be efficiently reversed by either substituting the cytoplasmic tail of GaLV Env with that from MuLV Env [12,13], deleting the GaLV Env R-peptide (carboxy-terminal half of the cytoplasmic domain) [13], or substituting key residues in the vicinity of the R-peptide cleavage site [13,14]. Furthermore, we observed that co-expression of lentiviral vector components led to decreased levels of GaLV Env in cells when compared to the expression levels observed in the presence of retroviral elements [13], suggesting a basis for this incompatibility

In the present study, we investigated the contribution of specific lentiviral vector components to the observed decrease in GaLV Env intracellular levels. Our results identified a major role for the HIV-1 Vpu protein which, interestingly, only occurred in the presence of other lentiviral packaging components. Similarities with the mechanism whereby Vpu degrades CD4 [15-20] and BST-2/tetherin [21-23] indicate that the GaLV Env protein may also contain features that make it a target for degradation by Vpu.

## Results

### GaLV TM subunit levels are decreased by lentiviral packaging constructs expressing HIV-1 accessory proteins

We have previously shown that levels of the GaLV Env trans-membrane (TM) subunit in cell lysates are strongly reduced in the presence of the lentiviral packaging plasmid pCMVΔR8.2 (R8.2) and that GaLV Env is unable to pseudotype lentiviral vector particles [13]. In contrast, we found that GaLV TM levels were unaffected by expression of the retroviral packaging plasmid pCgp, and that the GaLV Env was able to efficiently pseudotype retroviral vectors [13]. The GaLV Env protein is processed from a polypeptide precursor protein, Pr85, into SU and TM subunits that remain non-covalently linked in the viral particle [24]. The anti-TM antibody used in these studies did not detect Pr85, and in the absence of an anti-SU antibody, we were unable to distinguish between an effect of R8.2 co-expression on GaLV Pr85 stability, Pr85 processing to SU and TM, or TM stability.

**Figure 1:**
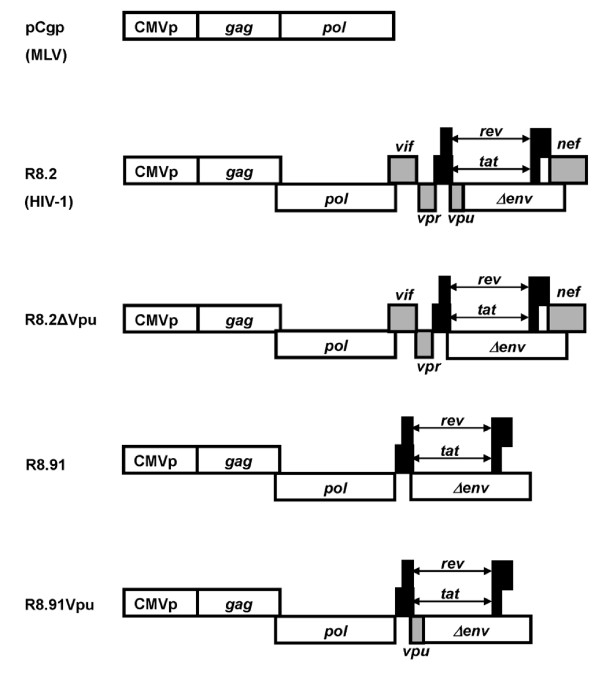
**Schematic representation of retroviral (pCgp) and lentiviral (R8.2, R8.2ΔVpu, R8.91 and R8.91Vpu) packaging constructs**. All lentiviral vectors express Gag, Pol, Tat and Rev; inclusion of Vif, Nef, Vpu and Vpr is as indicated (grey boxes).

**Figure 2:**
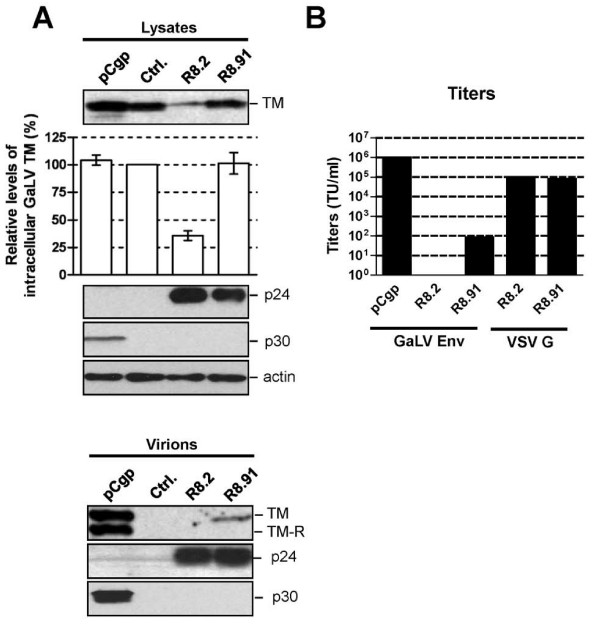
**GaLV Env levels and titers of pseudotyped vectors**. **A**. Western blot of a representative experiment showing levels of GaLV TM, HIV-1 p24, MLV p30 and actin in cell lysates and pelleted supernatants from 293T cells co-transfected with plasmids expressing GaLV Env, together with pCgp, R8.2, R8.91 or a control (Ctrl.) plasmid, as indicated. Also shown is a quantitative analysis of the steady-state levels of GaLV TM in cell lysates, made relative to the levels in the presence of control plasmid. Results are mean of three independent experiments. **B**. Titers of GaLV Env and VSV G pseudotyped retroviral (pCgp) and lentiviral (R8.2 and R8.91) vectors, as indicated, expressed as transducing units per ml (TU/ml). * indicates no detectable titer.

In order to identify the components of the lentiviral vector system responsible for these effects, we analyzed GaLV TM levels when lentiviral vectors were generated by the co-transfection of GaLV Env, a lentiviral vector transfer plasmid (pHR'-CMVLacZ), and the minimal lentiviral packaging construct, pCMVΔR8.91 (R8.91). R8.91 expresses HIV-1 Gag-Pol, Tat and Rev proteins, but does not express any of the HIV-1 accessory proteins (Vpu, Vif, Vpr or Nef) [25] (Figure 1). In contrast to the results observed in the presence of R8.2, which decreased the steady-state GaLV TM levels in transfected cells by approximately 60%, we found that co-expression of R8.91 had no effect on steady state GaLV TM levels in cell lysates (Figure 2A). This indicates that one or more accessory proteins of HIV-1 play a key role in the observed decrease in GaLV TM levels.

Interestingly, despite the lack of effect of R8.91 on intracellular GaLV TM levels, analysis of vector particles harvested from culture supernatants revealed a further defect in the incorporation of GaLV Env into R8.91 generated vector particles (Figure 2A). Although some TM protein could be detected in this fraction, it was present at considerably lower levels than in retroviral particles produced under the same conditions; and no mature, R-peptide cleaved form of the protein was apparent. Removal of the R peptide from the cytoplasmic tail of GaLV and MLV Env proteins by retroviral proteases activates their fusogenic potential and is necessary for full infectivity [26-28]. As expected, the GaLV Env pseudotyped vectors generated using R8.91 had very low titers (Figure 2B). As we have previously reported [13], no GaLV TM was detected in R8.2 derived lentiviral vector particles, and these vectors gave no titer. Taken together, these results suggest that two areas of incompatibility exist between the GaLV Env and lentiviral vectors. First, the expression of one or more HIV-1 accessory proteins in R8.2 reduces the intracellular steady-state levels of mature (cleaved) GaLV Env, while an additional block exists that reduces the incorporation and subsequent R-peptide processing of GaLV Env in lentiviral particles, even in the absence of any HIV-1 accessory proteins.

### Decrease in GaLV TM levels is mostly mediated by the HIV-1 Vpu protein

**Figure 3:**
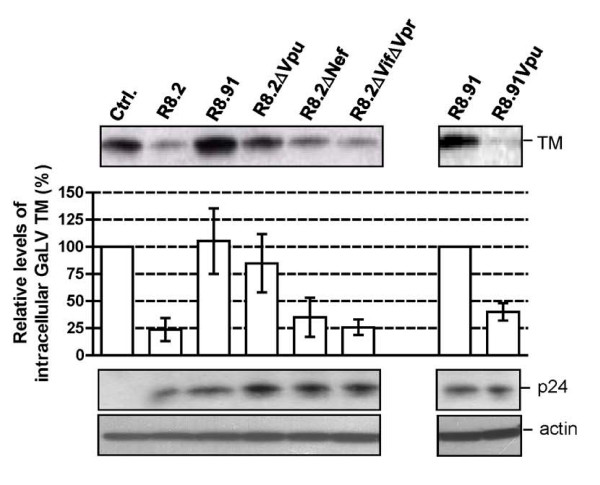
**Representative Western blot and quantitative analysis of signal density on blots from three independent experiments analyzing GaLV TM levels in lysates of 293T cells co-transfected with GaLV Env, pHR' and indicated derivatives of R8.2 and R8.91**. Immunological detection of cellular HIV-1 p24 and actin levels were included as controls.

In order to identify which of the HIV-1 accessory proteins expressed from R8.2 was responsible for the decrease in cellular levels of GaLV TM, we generated derivatives of R8.2 that were deleted for either Vpu, Nef, or a combination of the Vif and Vpr proteins, and co-expressed these plasmids with the lentiviral transfer vector and the GaLV Env. We found that the loss of Vpu in construct R8.2ΔVpu (Figure 1) had the greatest effect, resulting in only a 12% decrease in steady-state GaLV TM levels compared to the 60% inhibition that resulted from transfection of R8.2. In contrast, the absence of the Nef, Vpr or Vif proteins did not significantly stabilize the GaLV TM levels (Figure 3), although we did observe a consistently small enhancement in TM levels when Nef was deleted; but, overall, this was not statistically significant.

To confirm that Vpu was largely responsible for the effects on TM, we next added back the Vpu ORF into plasmid R8.91 (Figure 1). Expression of R8.91Vpu led to a marked decrease in GaLV TM levels, although not quite as complete as observed with R8.2. This finding also suggested a minor contribution to Env degradation from an additional HIV-1 accessory protein(s).

### Vpu alone is not sufficient to decrease GaLV TM levels

**Figure 4:**
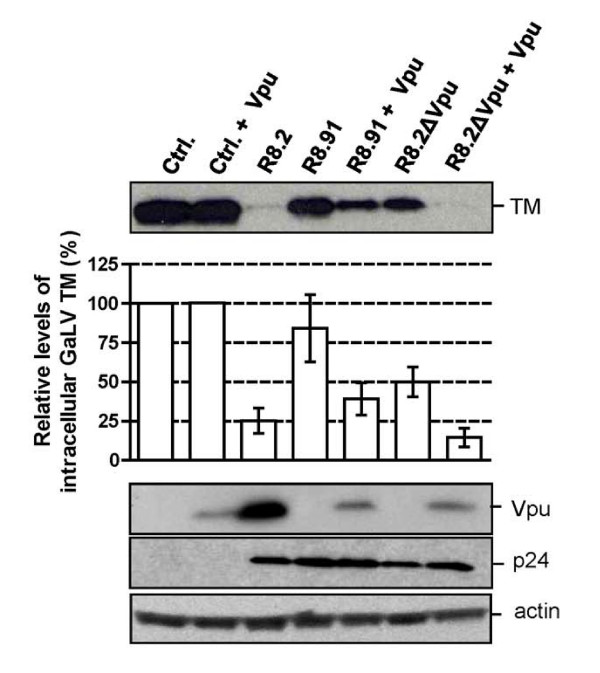
**Representative Western blot and quantitative analysis of signal density on blots from three independent experiments analyzing GaLV TM levels in lysates of 293T cells co-transfected with GaLV Env, pHR' and indicated derivatives of R8.2 or R8.91, with (+) expression of Vpu *in trans*, from plasmid CΔEvpu**. Immunological detection of HIV-1 p24 and actin levels were included as controls.

The ability of the Vpu protein to decrease steady state levels of GaLV TM was reminiscent of its known role in removing at least two cellular proteins, CD4 and BST-2/tetherin, from the cell surface [29,30]. Therefore, we next examined whether the effect on GaLV TM levels could be caused by expression of Vpu alone. Surprisingly, we observed that the co-transfection of GaLV Env and a Vpu expression plasmid (CΔEVpu) had no effect on GaLV TM levels (Figure 4). We noted that the level of expression of Vpu resulting from plasmid CΔEVpu was lower than from R8.2. However, we consider that this difference in Vpu expression levels is not likely to be the reason for the differences observed. This interpretation was based on the finding that the co-expression of CΔEVpu with R8.2ΔVpu caused equivalent reductions in TM levels as occurred with R8.2, despite the lower level of Vpu protein in the cells. In addition, we also investigated whether transfecting increasing amounts of the CΔEVpu plasmid, with a corresponding increase in the levels of Vpu, could reduce GaLV TM levels in the absence of a packaging plasmid, but we found that this was not the case (data not shown).

We next examined the consequences of co-expression of R8.91 and CΔEVpu, and found that this also resulted in a decrease in TM levels, in contrast to the results obtained with R8.91 or CΔEVpu alone. However, the more complete effect observed when the full set of HIV-1 genes was present, in the transfection of either R8.2, or R8.2ΔVpu plus CΔEVpu, suggests that an additional role is played by one or more of the other HIV-1 accessory proteins.

### Mutation of conserved serine residues in Vpu prevents the decrease in GaLV TM levels

**Figure 5:**
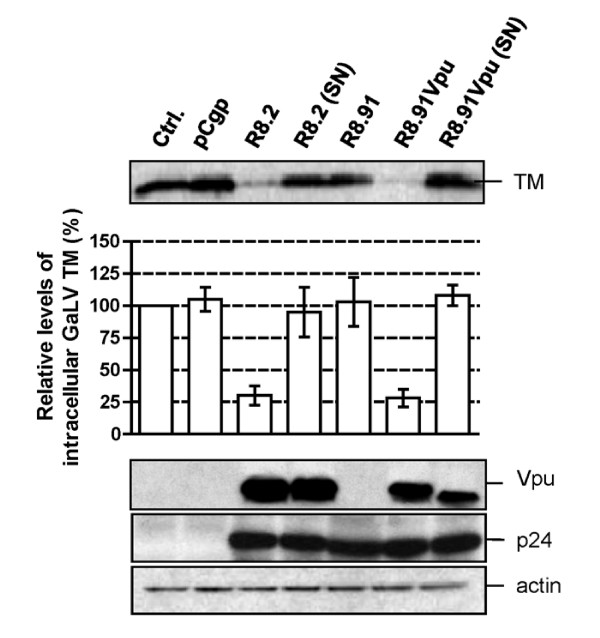
**Representative Western blot and quantitative analysis of signal density on blots from three independent experiments analyzing GaLV TM levels in lysates of 293T cells co-transfected with GaLV Env, pHR' and the indicated packaging plasmids**. (SN) indicates Vpu containing substitutions S52N and S56N. Immunological detection of HIV-1 p24 and cellular actin levels were included as controls.

Vpu targets CD4 for proteasomal degradation though recruitment of the SCF-E3 ubiquitin ligase complex [18,20]. Vpu binds to both CD4 and the β-TrCP subunit of the complex, with the interaction with β-TrCP requiring a conserved motif in Vpu's cytoplasmic tail that contains two phosphoserines (DS_52_GXXS_56_) [18,20]. Substitution of these serine residues with asparagine residues blocks CD4 degradation [15,16]. Recruitment of β-TrCP by Vpu has also been shown to play a role in counteracting the host budding restriction factor, BST-2/tetherin, and promoting either proteasomal [23] or lysosomal [21,22] degradation. We therefore introduced serine to asparagine substitutions into the packaging constructs R8.2 and R8.9.1Vpu, and analyzed their effects on GaLV TM levels. In both cases we observed that loss of the serines prevented the Vpu mediated decrease in GaLV TM levels (Figure 5). This suggests a role for the SCF-E3 ubiquitin ligase complex and protein degradation pathways in the reduction of GaLV TM levels.

### Substitution K618R in the GaLV tail confers resistance to R8.2

**Figure 6:**
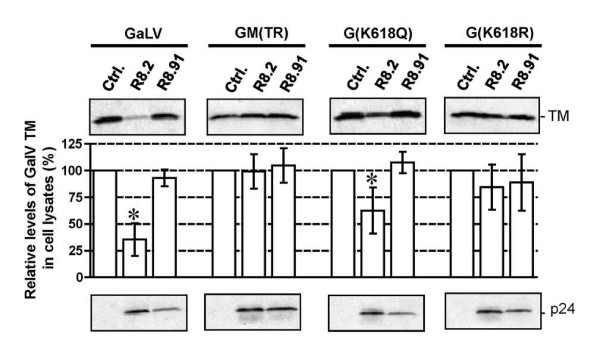
**Western blot and quantitative analysis of GaLV TM levels for wild-type and mutant versions of GaLV Env in the presence of pHR' and either control plasmid pGEM, or plasmids R8.2 or R8.91**. Graphs indicate relative levels of GaLV TM compared to the pGEM control, shown as the mean of 3-5 independent experiments. p-values were calculated, and statistical significance (p < 0.05) is indicated by an asterisk. HIV-1 p24 immunological detection was included as control.

We have previously shown that replacement of the cytoplasmic tail of GaLV Env with the equivalent domain from the MuLV Env prevents the decrease in TM levels resulting from expression of R8.2 [13]. In addition, we observed that this effect could be achieved by two GaLV to MuLV substitutions in the tail, K618Q and I619A [13]. Schubert et al. [19] previously reported that inhibition of the degradation of CD4 by Vpu occurred following the substitution of four lysines to arginines in CD4's cytosolic domain, suggesting ubiquitination as a mechanism. We therefore tested whether the loss of K618 alone was sufficient to preserve levels of GaLV TM by generating mutants K618Q and K618R. We found that the K618R substitution rendered the GaLV Env fully resistant to the R8.2-mediated decrease, similar to the results obtained when the whole of the GaLV Env tail was replaced with that of MuLV in construct GM(TR). In contrast, the K618Q substitution gave an intermediate phenotype (Figure 6).

### The SU subunit, but not the Pr85 precursor of GaLV Env, is affected by R8.2

**Figure 7:**
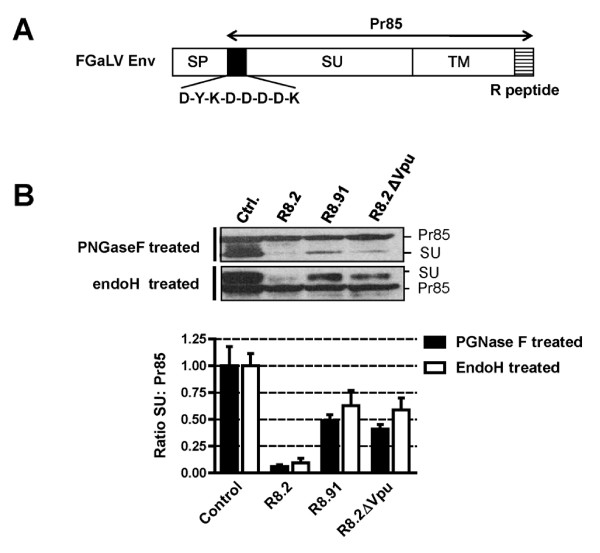
**Analyses of levels of GaLV Env Pr85 precursor and SU subunit**. **A**. Schematic representation of FLAG-tagged GaLV Env, showing precursor (Pr85), mature SU and TM subunits, signal peptide (SP) and R peptide (R). **B**. Representative Western blots and quantitative analysis of ratio of levels of FLAG-tagged GaLV Env Pr85 precursor and SU subunit, in presence of pHR' and either control plasmid pGEM, or packaging plasmids R8.2, R8.91 or R8.2ΔVpu. Samples were deglycosylated with either PGNase F or endoH prior to SDS-PAGE, and analyzed by immunoblotting with anti-FLAG antibody.

The GaLV Env protein is synthesized as an 85 kDa precursor (Pr85). This precursor protein is glycosylated in the ER and Golgi compartments into a short-lived 95 kDa intermediate that is present in the medial Golgi [31], and the protein is finally cleaved in the trans-Golgi network by a cellular furin protease into the mature SU (70 kDa) and TM (15 kDa) subunits. SU and TM remain non-covalently linked and are transported to the cell surface through the host vesicular transport system [24]. In an attempt to further understand the effect of R8.2 on GaLV Env, we investigated the fate of both the Pr85 and SU proteins in the presence of lentiviral vector components using a labeled version of the protein containing a FLAG tag at the N-terminus of SU (FGaLV Env) (Figure 7A). In addition, since the glycosylated forms of Pr85 and SU are not easily resolved by standard electrophoresis, we treated cells lysates with Peptide-N-glycosidase F (PNGaseF) in order to distinguish between the two Env proteins, as previously described [31,32]. Compared to control cells, we found that the SU subunit was barely detectable in the presence of R8.2, and was also significantly reduced by either R8.91 or R8.2ΔVpu, while none of the constructs had any obvious effect on the Pr85 levels (Figure 7B).

All or most of the carbohydrate chains that are added to glycoproteins in the ER or early Golgi become endoglycosidase H (endoH) resistant after passage through the medial Golgi, due to the action of cellular mannosidase I1 [33,34]. Bedgood and Stallcup have previously investigated the pattern of endoH-sensitivity in the closely related MuLV Env protein and found that the Pr85 precursor is endo H-sensitive, whereas the 70 kDa SU subunit and the transient 95 kDa intermediate it derives from comprise a mixture of endoH-sensitive and resistant species [31]. Using this assay to distinguish between Pr85 and SU, we found that deglycosylation of the various cell extracts with endoH yielded a similar pattern for the ratios of SU:Pr85 as was observed following PGNaseF treatment (Figure 7B). Specifically, Pr85 levels were unaffected while SU levels were inhibited by R8.2, and to a lesser extent by R8.91 or R8.2ΔVpu. Together these data suggest that the GaLV Env is targeted by both a Vpu-dependent mechanism, conferred by R8.2, as well as a Vpu- independent mechanism that can be provided by R8.91, and that both events occur after the SU-TM cleavage in the trans-Golgi.

### Vpu targets GaLV Env for degradation through the endolysosomal pathway

**Figure 8:**
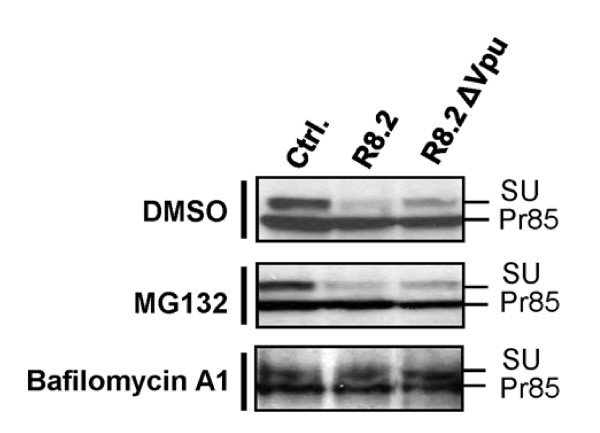
**Representative Western blots of levels of FLAG-tagged GaLV Env Pr85 precursor and SU subunit in presence of pHR' and either control plasmid pGEM (Ctrl.), R8.2 or R8.2ΔVpu**. Cells were treated with either the proteasomal inhibitor MG132, the lysosomal inhibitor bafilomycin A1, or DMSO. Samples were deglycosylated using endoH prior to SDS-PAGE, and analyzed by immunoblotting with anti-FLAG antibody.

Lastly, we investigated the mechanism for the decrease in GaLV SU levels caused by expression of R8.2 or R8.2ΔVpu (Figure 7B). Treatment of cells with either the proteasomal inhibitor MG132, or the lysosomal inhibitor bafilomycin A1, revealed that while inhibition of proteasomal degradation had no effect, SU levels were restored by treatment with bafilomycin A1 (Figure 8). Furthermore, this restoration occurred both in the absence and presence of Vpu. These findings suggest that both of the detrimental effects of lentiviral vectors on the GaLV Env are attributable to lysosomal degradation.

## Discussion

The ability of both retroviral and lentiviral vector particles to incorporate heterologous fusion proteins has been extensively exploited in the development of gene therapy vector systems [reviewed in [4] and [5]], as well as in studies of fusion proteins from biohazardous viruses [35-37]. Despite the general permissiveness of retroviral particles to accommodate unrelated fusion proteins, several examples exist where a lack of compatibility between vectors and fusion proteins occurs [12,13,38]. We have previously reported on the inability of the GaLV Env protein to pseudotype HIV-1 derived lentiviral vectors, where we observed a severe reduction in the levels of the mature GaLV TM subunit in cell lysates in the presence of lentiviral packaging plasmids [13]. In the present study we further investigated the basis for these reduced levels and identified a key role for the HIV-1 Vpu protein in this event. Studies using mutagenesis of both Vpu and GaLV Env, together with results obtained using chemical inhibitors of cellular degradation pathways, suggested a ubiquitin-dependent targeting of GaLV Env to lysosomal degradation, occurring at a stage in GaLV Env trafficking that is at, or beyond, the trans-Golgi network.

The HIV-1 Vpu protein is known to have several different activities [39]. Its cytoplasmic tail contains a canonical DSGXXS destruction motif that recruits β- TrCP, a component of the SCF E3 ubiquitin ligase complex, and thereby mediates the degradation of at least two host cell proteins, CD4 and BST-2/tetherin [18,20-22]. Simultaneous binding of Vpu to the cytoplasmic tail of CD4 and the SCF ubiquitin ligase complex targets the protein for proteasomal degradation [40-43], while the downregulation of BST-2/tetherin, has been suggested to involve either proteasomal [23] or lysosomal [21,22] pathways.

Binding of Vpu to β- TrCP is critically dependent on the phosphorylation of the two conserved serines, S52 and S56, in the DSGXXS motif [15,16]. We found that these residues were also essential for Vpu's activity against the GaLV Env, suggesting that a similar β- TrCP-dependent mechanism is involved. Vpu targeted the GaLV Env at a stage after cleavage of the Env precursor, Pr85, into SU and TM subunits, suggesting that the site of action was the trans-Golgi network or beyond. Lastly, we showed that Vpu-mediated degradation of GaLV Env was achieved through the lysosomal, not proteasomal pathway.

In addition, we observed that the substitution K618R in the GaLV Env cytoplasmic tail completely blocked the Vpu-dependent effect on TM levels. Although this could suggest a role for ubiquitination at this lysine residue, the K618Q substitution resulted in only a partially rescued phenotype; thus, ubiquitination at this specific residue may not be essential for the effect.

In addition to the strong phenotype associated with the presence or absence of the Vpu protein, our study also revealed that more than one level of incompatibility exists between lentiviral vectors and GaLV Env. Even when Vpu was not expressed, lentiviral vectors resulting from the co-expression of GaLV Env and R8.91 incorporated only very low levels of Env, with no detectable cleavage of the R-peptide observed, and the resulting titers were extremely low. Other examples of incompatibility between Envs and particles have been described within the *Retroviridae*, including a block to incorporation of the Env protein from feline endogenous virus RD114 into lentiviral vectors based on simian immunodeficiency virus (SIV) [38], which was suggested to be caused by a lack of Gag-Env interaction/co-localization [44,45]. Although beyond the scope of this study, it will be interesting to determine if such a mismatch in Env-Gag interaction and/or localization underlies the low-level incorporation and titers observed for GaLV Env pseudotyped lentiviral vectors based on R8.91.

An especially interesting aspect of this study was the finding that Vpu alone was not sufficient to degrade GaLV Env and block its incorporation into lentiviral vectors, but this observation additionally required the presence of one or more of the HIV-1 proteins present on R8.91 (Gag-Pol, Tat, Rev) and to an even greater extent in R8.2ΔVpu (additionally expresses Vif, Vpr and Nef). Several possible mechanisms can be envisioned to explain these requirements. First, Vpu itself could be altered in some way by the presence of another HIV-1 component, either directly or indirectly, and this modification could be required to target the GaLV Env. Alternatively, the ability of Vpu to target GaLV Env may require a co-localization of the two proteins, in a manner that could be promoted by the assembly and/or budding of HIV-1 particles in the same cell. Such a mechanism could involve either recruitment of Vpu to sites where the GaLV Env is also present or, alternatively, the recruitment of the GaLV Env to sites where it becomes Vpu-accessible. Targeting of the CD4 receptor by Vpu occurs in the ER [46], while Vpu appears to target BST-2/tetherin in the late endosomes [22]. Our data suggest that since only the steady-state levels of SU and TM, and not Pr85, were decreased, Vpu is likely to target GaLV Env at or beyond the stage of Pr85 cleavage by furin in the trans-Golgi network. It is also possible that Vpu interferes with transport of Pr85 through the ER and Golgi compartments, although such an effect would be expected to result in the intracellular accumulation of Pr85, and perhaps the 95 kDa intermediate, which we did not observe.

Since the reduction in GaLV TM levels caused by R8.2, or the co-expression of R8.2ΔVpu plus Vpu in *trans*, was consistently greater than that achieved with R8.91 plus Vpu in *trans*, the involvement of another HIV-1 accessory protein in the overall effect on the GaLV Env is also indicated. Indeed, a role for the HIV-1 Nef protein was suggested by the small but consistent differences in activity for R82ΔNef compared to R8.2, although the lack of statistical significance requires caution in the interpretation of these data. It has been previously shown that expression of Nef re-directs the RD114 Env to late endosomes [45]. Since Vpu has been shown to target BST-2/tetherin in late endosomes [22], it is possible that a minor cooperative effect exists between these two HIV-1 accessory proteins. Alternatively, since Nef has previously been shown to mediate the endocytosis and rapid degradation of cell surface CD4 in lysosomes [47,48], it is also possible that a minor part of the effect seen on GaLV Env levels could be directly attributable to a similar activity of Nef.

## Conclusions

The destabilization of the GaLV Env protein that occurs in the presence of lentiviral vector components is mostly attributable to the action of the HIV-1 Vpu protein. Our data suggest that this event involves the recruitment of the SCF E3 ubiquitin ligase complex and the targeting of protein for lysosomal degradation, as previously demonstrated for BST-2/tetherin down-modulation by Vpu. However, degradation of the GaLV Env by Vpu is a distinct event in that it additionally requires the presence of other, as yet unidentified, HIV-1 components. Although the exact mechanism involved is not yet clear, these observations suggest that a more complex interaction may exist between Vpu and its target proteins, that is regulated by some other aspect of HIV-1 replication, and which provide an extra level of control that has not previously been appreciated. Since HIV-1 and GaLV do not infect the same hosts, it is most likely that this targeting of the GaLV Env by HIV-1 reflects some essential similarity in the GaLV Env protein to other host or viral proteins that are *bona fide *targets of Vpu's activity.

## Materials and methods

### Cell lines

Human kidney epithelial 293T cells (ATCC, Manassas, VA) were maintained in D10, Dulbecco's modified Eagle's medium (Mediatech, Herndon, VA) supplemented with 10% fetal bovine serum (Hyclone, Logan, UT) and 2 mM glutamine (Gemini Bio-Products, West Sacramento, CA).

### Plasmids

All GaLV Env proteins were based on the SEATO strain (Genbank: NP056791) and expressed from plasmids containing the human cytomegalovirus (CMV) immediate early promoter. Construct GM(TR) is a GaLV Env containing the cytoplasmic tail of the amphotropic MLV Env and has been described previously [13]. The GaLV Env mutants K618R and K618Q were generated by PCR-directed mutagenesis. The FGaLV Env construct was generated by substituting the 5'-terminal sequence of GaLV Env ORF in our expression plasmid by that of the FLAG-tagged FGaLV.fus [49], kindly donated by Dr Adele Fielding. Retroviral packaging plasmid pCgp that expresses MLV Gag-Pol, and transfer vector pCnBg, have been described previously [50]. HIV-1 packaging constructs pCMVΔR8.2 and pCMVΔR8.91 (herein abbreviated as R8.2 and R8.91) and transfer vector pHR'-CMVLacZ have been described previously [1,25,51]. Constructs R8.2ΔVpu, R8.2ΔVifΔVpr and R8.2ΔNef were created as chimeras between R8.2 and R8.91 by inserting SalI- BamHI, SwaI-SalI and BamHI-XbaI fragments, respectively, from R8.2 into R8.91. Plasmid R8.91Vpu was created by insertion of BamHI-SalI fragment from R8.2 into R8.91. Constructs R8.2Vpu(SN) and R8.91(SN) contain S to N substitutions at residues 52 and 56 in the Vpu protein and were created by PCR mutagenesis The CΔEVpu vector expresses Vpu under the control of the CMV promoter, and the parental expression plasmid CΔE (no ORF) was used as a control plasmid to ensure equivalent levels of DNA in transfections.

### Production of retroviral and lentiviral vectors

Retroviral vectors were generated by the co-transfection of plasmids pCgp, pCnBg and a plasmid encoding an appropriate GaLV Env protein into 293T cells, essentially as described [13,50], with culture supernatants containing vector particles harvested 24 hours post transfection. Lentiviral vectors were generated in the same way using packaging plasmids R8.2 or R8.91 and in the presence of transfer genome plasmid pHR'-CMVLacZ [13,50]. Control transfections included equivalent amounts of cloning vector plasmid pGEM (Promega, Madison, WI).

### Western blotting of viral vector particles

Vector particles were harvested from the supernatants of transiently transfected 293T cells and partially purified by centrifugation through 2 ml of 20% sucrose at 75,000 × g. at 4°C for 2 hours. Cell monolayers of transfected 293T cells were incubated in 200 μl lysis buffer (20 mM Tris-HCl, pH 7.5, 1% triton X-100, 0.05% SDS, 5 mg/ml sodium deoxycholate, 150 mM NaCl, and 1 mM phenylmethanesulphonylfluoride) for 10 min at 4°C, followed by centrifugation at 10,000 × g for 10 minutes to pellet cell debris. All protein samples were normalized for equal protein concentration using the Bradford assay (Biorad, Hercules, CA) prior to electrophoresis and Western blotting. The transmembrane (TM) subunit of the GaLV Env was detected with rat monoclonal antibody 42/114 [52]. Vpu was detected with the HIV-1_NL4-3 _Vpu antiserum [53], p24 with the HIV-1_SF2 _p24 rabbit antiserum, both obtained through the AIDS Research and Reference Reagent Program, Division of AIDS, NIAID, NIH. FLAG-tagged GaLV Env protein was detected using mouse M2 anti-FLAG antibody (Sigma, St Louis, MO). Actin was detected using anti-actin monoclonal antibody (clone C4, Roche Applied Science, Indianapolis, IN), and MuLV p30 using the goat anti-Rauscher MLV CA antiserum (Quality Biotech, Camden, NJ).

### Analysis of glycosylated proteins

Fifteen μl of cell lysates were heated at 95°C for 5 minutes, in peptide N-Glycosidase F (PNGaseF) denaturation buffer, followed by the addition of 1× G7 buffer, 1% NP40 and 500 U PNGaseF (NEB, Ipswich, MA), and samples were incubated at 37°C for 1 hour. Alternatively, 15 μl of cell lysates were heated at 100°C for 10 minutes in 1× endoglycosidase H (endoH) denaturation buffer, followed by the addition of 1× G5 buffer and 500 U of endoH (NEB, Ipswich, MA), and samples incubated at 37°C for 1 hour. Treated lysates were resolved by SDS-PAGE, followed by Western blotting using anti-FLAG antibody.

### Inhibitor studies

Proteasomal or lysosomal degradation pathways were inhibited by incubation with MG132 and bafilomycin A1 (Sigma, St Louis, MO) at concentrations of 10 μM and 100 nM respectively, for 8 hours.

## Competing interests

The authors declare that they have no competing interests.

## Authors' contributions

IC carried out most of the experimental work and participated in the analysis of results, MEDB participated in the experimental work, interpretation of data and writing the manuscript, JEO participated in the experimental work, PMC conceived the study, participated in its design and co-ordination and helped to write the manuscript. All authors read and approved the final manuscript.
